# Serum manganese and its association with non-alcoholic fatty liver disease: findings from NHANES

**DOI:** 10.3389/fnut.2025.1527207

**Published:** 2025-03-28

**Authors:** Zipeng He, Yanrui Zhao, Hua Tang

**Affiliations:** ^1^Department of Ultrasound Medicine, Beijing Chao-Yang Hospital, Capital Medical University, Beijing, China; ^2^Department of Radiology, The First Hospital of Fangshan Distict, Beijing, China

**Keywords:** serum manganese, non-alcoholic fatty liver disease, transient elastography of liver, body mass index, hypertension, ethnicity, gender

## Abstract

**Objective:**

This study examines the link between serum manganese (Mn) levels and non-alcoholic fatty liver disease (NAFLD), with a focus on gender differences.

**Methods:**

Utilizing data from the NHANES 2017–2018, we included participants aged 18 and older, excluding those without ultrasonic liver assessment, serum Mn data, or with hepatitis or significant alcohol use. The final analysis comprised 4,294 individuals, with 2,708 in the NAFLD group and 1,586 in the non-NAFLD group. Serum Mn was quantified via inductively coupled plasma mass spectrometry. We compared demographic and health-related variables between groups using appropriate statistical tests and categorized participants into quartiles based on Mn levels. Multivariate logistic regression and spline regression analyses were conducted to evaluate the association between serum Mn and NAFLD risk by gender.

**Results:**

Serum Mn was significantly elevated in the NAFLD group compared to non-NAFLD individuals (9.06 vs. 9.33 μg/L, Z = 2.815, *p* = 0.005). After adjustments, males in the third Mn quartile showed a higher NAFLD risk (OR = 1.575; 95% CI: 1.193–2.087), while females in the fourth quartile also had increased risk (OR = 1.725; 95% CI: 1.313–2.269), both compared to the first quartile (*p* < 0.01). A positive dose–response relationship was found for both genders (*P* for trend <0.01), with nonlinear associations in males (*P* for nonlinearity <0.01) and linear associations in females (*P* for nonlinearity = 0.818). Significant interactions with ethnicity in males and hypertension in females were also noted.

**Conclusion:**

Higher serum Mn levels are significantly associated with increased NAFLD risk in both genders, highlighting the need for gender-specific considerations in future studies and clinical practices.

## Introduction

Non-alcoholic fatty liver disease (NAFLD) is a clinical-pathological syndrome characterized by diffuse macrovesicular fatty degeneration and lipid accumulation in hepatocytes, primarily affecting the liver lobule. Unlike other liver diseases, NAFLD is not attributable to alcohol consumption or other clearly hepatotoxic factors (1). Epidemiological surveys have highlighted the growing prevalence of NAFLD, which stands at 25.24% globally, making it the most common chronic liver condition worldwide (2). The increasing incidence of NAFLD is closely linked to rising obesity rates, influenced by improved living standards, dietary changes, sedentary lifestyles, and environmental contaminants, which have established etiologic roles with strong sex-dimorphism (3, 4). Projections suggest that by 2030, the number of NAFLD cases will escalate by 21%, reaching 100.9 million from 83.1 million in 2015 (5). If left unmanaged, NAFLD can progress to more severe liver conditions, such as fibrosis, cirrhosis, and hepatocellular carcinoma (6).

Manganese (Mn) is a trace element with toxic potential, which enters the body mainly through the gastrointestinal tract from sources such as dietary intake, including the consumption of vegetables and fruits contaminated with fungicides such as maneb and mancozeb, as well as other environmental exposures (7). Mn is crucial for the clearance of reactive oxygen species (ROS) from mitochondrial oxidative stress, primarily through its role in the enzyme manganese superoxide dismutase (Mn-SOD). The C47T polymorphism in the SOD2 gene, which affects Mn-SOD’s mitochondrial targeting and activity, has been identified as an independent risk factor for advanced fibrosis in NAFLD (8). Although the exact pathogenesis of NAFLD remains elusive, the involvement of ROS, oxidative stress, inflammation, and fatty acid metabolism imbalances are key contributing factors (9). Elevated serum Mn levels can exacerbate NAFLD progression by influencing fat accumulation, lipogenesis, insulin resistance, oxidative stress, and inflammation (10).

With the modernization of agriculture and industry, metal pollution has intensified, leading to a growing concern about the impact of various metals on health. Studies have demonstrated significant associations between NAFLD and exposures to metals such as cadmium (11) and arsenic (12), among others (13). Despite the lack of safe and effective treatments for NAFLD, research on the correlation between trace metal Mn and NAFLD is still limited. Notably, serum Mn levels exhibit significant gender differences, with females showing higher levels than males (14).

However, it is important to note that serum Mn levels may not be fully predictive of internal contamination. Studies suggest that matrices like hair could provide a more accurate assessment of Mn exposure due to its longer retention time and less fluctuation compared to serum levels (14). This limitation should be considered when interpreting the results of this study.

This study aims to analyze these gender differences separately, exploring the correlation between serum Mn levels and the risk of NAFLD progression in both males and females. By doing so, the study seeks to enhance the understanding of NAFLD pathogenesis from different perspectives and identify potential biomarkers for its development. This could ultimately aid in formulating effective prevention strategies at both the individual and population levels.

## Participants and methods

### Participants

The National Health and Nutrition Examination Survey (NHANES) is a comprehensive cross-sectional study aimed at evaluating the health and nutritional status of the U.S. population (15). Conducted every 2 years, NHANES gathers extensive data encompassing demographic, lifestyle, health, and nutritional information from participants. The NHANES public database can be accessed at NHANES CDC.^1^ For this investigation, we utilized data from the 2017–2018 NHANES cycle. Our inclusion criteria focused on registered participants aged 18 years and older, yielding an initial cohort of 5,856 individuals. We applied several exclusion criteria: (1) absence of liver ultrasound transient elastography results (*n* = 253); (2) diagnosis of hepatitis B or C (*n* = 85); (3) significant alcohol intake (men >30 g/day, women >20 g/day) (*n* = 478); (4) missing critical laboratory data such as alanine aminotransferase (ALT) and aspartate aminotransferase (AST) (*n* = 489); and (5) unavailability of serum manganese (Mn) levels (*n* = 257). Ultimately, a total of 4,294 participants were retained for analysis.

### Assessment of NAFLD

Liver ultrasound transient elastography serves as a non-invasive, objective method for diagnosing non-alcoholic fatty liver disease (NAFLD), noted for its robust sensitivity and specificity in population studies (16). The Controlled Attenuation Parameter (CAP) is a key indicator for NAFLD detection, with performance comparable to that of liver biopsy, which is considered the gold standard. A diagnosis of NAFLD is established with a CAP value of 223 dB/m or above, while excluding individuals with hepatitis B, hepatitis C, autoimmune liver disorders, and significant alcohol use (men >30 g/day, women >20 g/day) (17).

### Serum Mn levels

Serum manganese levels were measured at the Environmental Health Sciences Laboratory of the National Center for Environmental Health using inductively coupled plasma dynamic reaction cell mass spectrometry. This process adheres to rigorous quality control standards (18). Normal serum Mn concentrations typically range from 4 to 15 μg/L. (19) In this study, the detection threshold for serum Mn was set at 0.990 μg/L, with any values below this limit substituted with the detection limit divided by the square root of 2.

### Statistical methods

We conducted statistical analyses using R version 4.2.2. For data that exhibited skewed distributions, results are presented as medians (M) with interquartile ranges (P25, P75), and comparisons were performed using the Wilcoxon rank-sum test. Categorical variables were represented as counts and percentages, with differences assessed via the chi-square (χ^2^) test. To explore the association between serum Mn levels and NAFLD, we employed multivariate adjusted logistic regression models. Serum Mn levels were analyzed both as continuous and categorical variables, stratified into quartiles with the first quartile serving as the reference group. Odds ratios (OR) and 95% confidence intervals (CI) were computed across three modeling approaches: Model 1, which included no adjustments; Model 2, which adjusted for demographic factors such as age, ethnicity, education, marital status, Family-to-Poverty Ratio (FMPIR), and Body Mass Index (BMI); and Model 3, which further adjusted for health-related factors including smoking, alcohol consumption, diabetes, hypertension, and hyperlipidemia.

We also utilized restricted cubic spline regression to examine non-linear relationships between serum Mn levels and NAFLD, visualizing the dose–response association. Additionally, subgroup analyses were performed, categorizing participants by age, ethnicity, education, marital status, FMPIR, smoking status, alcohol consumption, and history of diabetes, hypertension, and hyperlipidemia. Interaction terms were incorporated into our models, and likelihood ratio tests were conducted to assess the presence of interactions, thereby uncovering potential variations in the relationship between serum Mn levels and NAFLD. A *p*-value of less than 0.05 was deemed statistically significant.

## Results

### Demographic characteristics of study participants

The study comprised 4,294 participants, divided into 2,708 individuals with NAFLD and 1,586 without NAFLD. Notable differences in demographic and clinical features were evident between these groups. Participants with NAFLD were significantly older and included a higher proportion of males, Mexican Americans, individuals with high school education or less, and those with a history of smoking more than 100 cigarettes in their lifetime. Additionally, the NAFLD group had more married individuals or those living with a partner, and a larger proportion fell within the FMPIR range of 1.30 to 3.50 and had a BMI of 30 or higher.

Clinically, the NAFLD group exhibited elevated levels of systolic and diastolic blood pressure, HOMA-IR, waist circumference, triglycerides, ALT, AST, GGT, fasting glucose, CRP, HbA1c, and CAP, along with reduced levels of HDL-C. The prevalence of diabetes, hypertension, and hyperlipidemia was also significantly higher in the NAFLD group (*p* < 0.01). Furthermore, serum manganese (Mn) levels were higher in the NAFLD group compared to the non-NAFLD group. Detailed demographic and clinical data are presented in Table 1.

**Table 1:** Basic characteristics of research subjects.

Characteristics	Non-NAFLD Group (*n* = 1,586)	NAFLD Group (*n* = 2,708)	*z*/*χ*^2^ value	*p* value
Age (Years)^a^	41 (27, 61)	55 (40, 66)	13.899	<0.001
Age Group^b^				
18–39 Years	755 (47.60)	643 (23.74)		
40-59 Years	370 (23.33)	917 (33.86)	159.321	<0.001
≥ 60 Years	461 (29.07)	1,148 (42.39)		
Gender^b^				
Female	924 (58.26)	1,336 (49.34)		
Male	662 (41.74)	1,372 (50.66)	31.954	<0.001
Ethnicity^b^				
Mexican American	154 (9.71)	447 (16.51)		
Non-Hispanic Black	428 (26.99)	540 (19.94)	55.251	<0.001
Non-Hispanic White	548 (34.55)	954 (35.23)		
Other	456 (28.75)	767 (28.32)		
Education^b^				
College or Above	939 (59.21)	1,511 (55.80)		
High School	378 (23.83)	654 (24.15)	7.131	0.028
Below High School	269 (16.96)	543 (20.05)		
Lifetime Smoking Number^b^				
< 100 Cigarettes	995 (62.74)	1,577 (58.24)		
≥ 100 Cigarettes	591 (37.26)	1,131 (41.76)	8.438	0.004
Marital Status^b^				
Married/Living with Partner	807 (50.88)	1,656 (61.15)		
Never Married	453 (28.56)	428 (15.80)	100.530	<0.001
Widowed/Divorced/Separated	326 (20.56)	624 (23.04)		
Alcohol Consumption^a^	1.392 (87.77)	2,409 (88.96)	1.395	0.258
FMPIR^a^	2.03 (1.10, 3.98)	2.19 (1.22, 4.17)	2.404	0.016
FMPIR Grouping^b^				
<1.30	504 (31.78)	741 (27.36)		
≥3.50	475 (29.95)	846 (31.24)	9.690	0.008
1.30 ≤ FMPIR < 3.50	607 (38.27)	1,121 (41.40)		
BMI (kg/m^2^)^a^	24.7 (21.7,28.0)	30.4 (26.8, 35.2)	12.879	<0.001
BMIGrouping^b^				
< 25 kg/m^2^	823 (51.89)	376 (13.88)		
≥ 30 kg/m^2^	275 (17.34)	1,429 (52.77)	835.881	<0.001
25 ~ <30 kg/m^2^	488 (30.77)	903 (33.35)		
Systolic Blood Pressure (mmHg)^a^	117 (107,130)	125 (115,136)	13.976	<0.001
Diastolic Blood Pressure (mmHg)^a^	70 (63, 77)	73 (66, 81)	9.425	<0.001
HOMA-IR^a^	1.73 (1.11, 2.68)	3.14 (2.03, 4.98)	26.577	<0.001
WC(cm)^a^	87.20 (78.70, 96.80)	104.20 (95.10,115.70)	33.555	<0.001
TG (mmol/L)^a^	1.00 (0.73, 1.42)	1.47 (1.05, 2.03)	22.057	<0.001
ALT (μ/L)^a^	14 (11, 19)	18 (14, 25)	16.639	<0.001
AST (μ/L)^a^	18 (15, 21)	19 (16, 23)	4.680	<0.001
GGT (IU/L)^a^	16 (12, 22)	21 (16, 29)	17.662	<0.001
HDL-C (mmol/L)^a^	1.45 (1.24, 1.71)	1.24 (1.06, 1.50)	17.203	<0.001
GLU (mmol/L)^a^	5.50 (5.16, 5.88)	5.94 (5.50, 6.66)	20.859	<0.001
CRP (mg/L)^a^	1.14 (0.60, 2.78)	2.31 (1.08, 4.72)	16.532	<0.001
HbA1c (%)^a^	5.40 (5.20, 5.70)	5.70 (5.40, 6.10)	18.207	<0.001
CAP (db/m)^a^	205 (182, 221)	292 (265, 327)	54.772	<0.001
Hypertension^b^	173 (10.91)	489 (18.06)	39.209	<0.001
Hyperlipidemia^a^	60 (3.78)	370 (13.66)	108.331	<0.001
Diabetes^b^	95 (5.99)	526 (19.42)	145.919	<0.001
Serum Mn (μg/L)^a^	9.06 (7.25, 11.38)	9.33 (7.54, 11.52)	2.815	0.005

a Data are presented as M (P25, P75).

b Data are presented as cases (%).

### Logistic analysis of serum Mn and NAFLD by gender

To determine the association between serum Mn levels and NAFLD, multivariate logistic regression models were employed, stratified by gender. Serum Mn was assessed both as a continuous variable and across quartiles.

For males, each quartile increase in serum Mn was associated with a 25.20% (OR = 1.252, 95% CI: 1.097–1.429), 17.70% (OR = 1.177, 95% CI: 1.020–1.358), and 18.90% (OR = 1.189, 95% CI: 1.028–1.375) higher risk of NAFLD in models 1, 2, and 3, respectively. When analyzed by quartiles, in model 3, the risk of NAFLD increased by 40.70% (OR = 1.407, 95% CI: 1.097–1.807) and 57.50% (OR = 1.575, 95% CI: 1.193–2.087) in the Q2 and Q3 groups compared to the lowest Mn group (Q1).

For females, similar trends were observed. The risk of NAFLD increased by 12.90% (OR = 1.129, 95% CI: 1.018–1.252), 26.60% (OR = 1.266, 95% CI: 1.128–1.421), and 32.40% (OR = 1.324, 95% CI: 1.176–1.491) per quartile increase in serum Mn in models 1, 2, and 3, respectively. When comparing quartiles in model 3, the risk of NAFLD was higher by 30.60% (OR = 1.306, 95% CI: 1.006–1.696), 44.40% (OR = 1.444, 95% CI: 1.109–1.882), and 72.50% (OR = 1.725, 95% CI: 1.313–2.269) in the Q2, Q3, and Q4 groups, respectively, relative to the lowest Mn group (Q1).

These findings highlight a significant association between elevated serum Mn levels and increased risk of NAFLD, with varying degrees of risk observed across different quartiles of Mn concentration. Detailed logistic regression results are provided in Tables 2, 3.

**Table 2:** Logistic analysis of serum Mn and NAFLD in male.

Group	Model 1		Model 2		Model 3	
	OR(95%CI)	*p* value	OR(95%CI)	*p* value	OR(95%CI)	*p* value
Mn	1.252 (1.097,1.429)	0.001	1.177 (1.020,1.358)	0.026	1.189 (1.028–1.375)	0.020
Q1 Group	1.000	-	1.000	-	1.000	-
Q2 Group	1.468 (1.164, 1.854)	0.001	1.374 (1.074, 1.759)	0.012	1.407 (1.097–1.807)	0.007
Q3 Group	1.714 (1.325, 2.227)	<0.001	1.573 (1.196, 2.076)	0.002	1.575 (1.193–2.087)	0.001
Q4 Group	1.325 (0.995, 1.772)	0.056	1.159 (0.851, 1.585)	0.352	1.180 (0.861–1.624)	0.306

**Table 3:** Logistic analysis of serum Mn and NAFLD in female.

Group	Model 1		Model 2		Model 3	
	OR(95%CI)	*p* value	OR(95%CI)	*p* value	OR(95%CI)	*p* value
Mn	1.129 (1.018, 1.252)	0.022	1.266 (1.128, 1.421)	<0.001	1.324 (1.176–1.491)	<0.001
Q1 Group	1.000	—	1.000	—	1.000	—
Q2 Group	1.147 (0.903, 1.458)	0.261	1.243 (0.964, 1.603)	0.094	1.306 (1.006–1.696)	0.045
Q3 Group	1.172 (0.922, 1.490)	0.195	1.367 (1.057, 1.769)	0.017	1.444 (1.109–1.882)	0.006
Q4 Group	1.254 (0.986, 1.595)	0.065	1.563 (1.198, 2.040)	0.001	1.725 (1.313–2.269)	<0.001

Model 1: No confounders adjusted. Model 2: Adjusted for sociodemographic variables including age, ethnicity, education, marital status, FMPIR, and BMI. Model 3: Further adjusted for health-related variables including smoking, alcohol consumption, diabetes, hypertension, and hyperlipidemia on the basis of Model 2. —: No value available.

### Dose–response relationship between serum manganese (Mn) and NAFLD by gender

After adjusting for variables such as age, ethnicity, education, marital status, FMPIR, BMI, smoking, alcohol consumption, diabetes, hypertension, and hyperlipidemia, we employed restricted cubic spline regression analysis to examine the relationship between serum manganese (Mn) levels and the risk of non-alcoholic fatty liver disease (NAFLD) by gender.

In males, a positive dose–response relationship was observed between serum Mn levels and the risk of NAFLD (*p* < 0.01), with a significant non-linear component (P for nonlinearity <0.01). Specifically, serum Mn levels below 8.747 μg/L were protective against NAFLD, with the protective effect diminishing as Mn levels increased. Between 8.747 μg/L and 10.909 μg/L, serum Mn levels were associated with an increased risk of NAFLD, and this risk continued to rise with higher levels. Beyond 10.909 μg/L, no significant association with NAFLD risk was detected.

In females, the positive dose–response relationship between serum Mn levels and NAFLD risk was also significant (*p* < 0.01), but the relationship was linear (P for nonlinearity = 0.818). Serum Mn levels below 9.850 μg/L were protective, with the protective effect declining as levels increased. When serum Mn levels exceeded 9.850 μg/L, the risk of NAFLD increased significantly.

**Figure 1:**
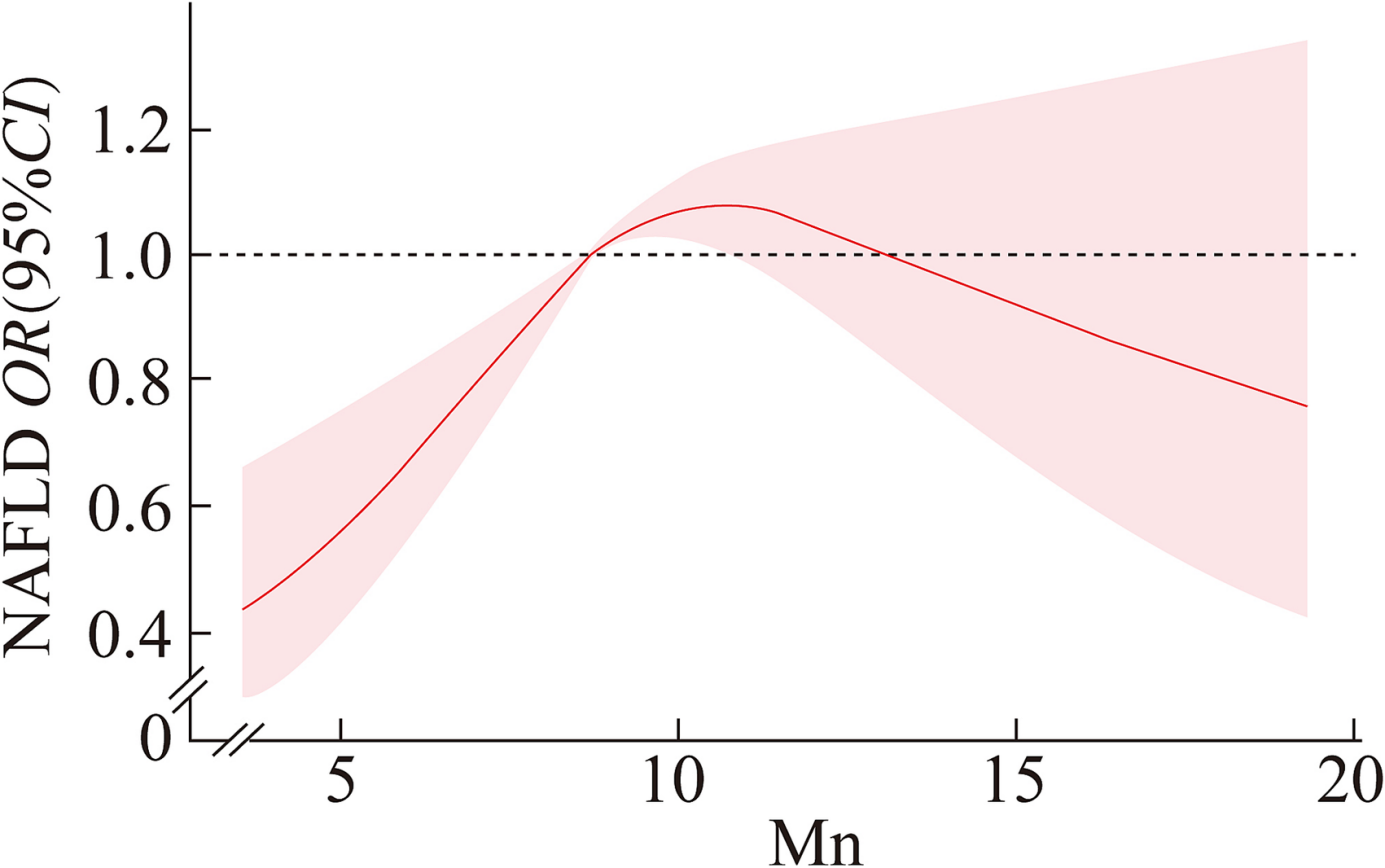
Dose–response relationship between serum Mn and NAFLD in male.

**Figure 2:**
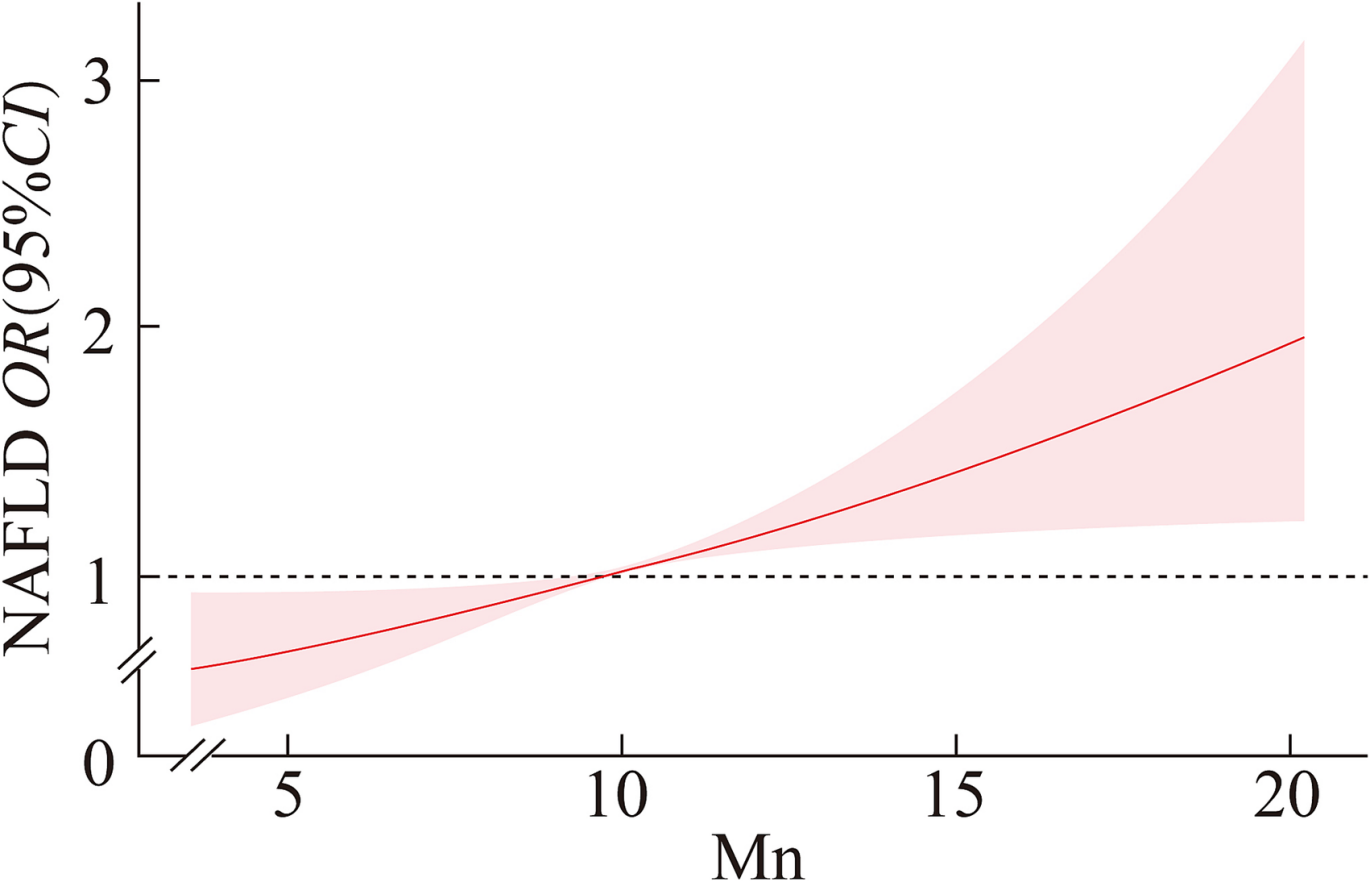
Dose–response relationship between serum Mn and NAFLD in female.

Figures 1, 2 provide a detailed illustration of these relationships.

### Subgroup analysis of serum Mn and NAFLD by gender

In males, significant interactions were found between serum Mn levels and both ethnicity (*p* = 0.011) and education (*p* = 0.020). For non-Hispanic Black males, each quartile increase in serum Mn was associated with a 52.90% increase in the risk of NAFLD (OR = 1.529; 95% CI: 1.164–2.008). Additionally, males with education below high school experienced a 70.30% increase in NAFLD risk (OR = 1.703; 95% CI: 1.233–2.351), with these differences being statistically significant (*p* < 0.01).

In females, a significant interaction was observed between serum Mn levels and hypertension status (*p* = 0.006). Hypertensive females experienced a 65.80% increase in NAFLD risk (OR = 1.658; 95% CI: 1.153–2.386) with each quartile increase in serum Mn. Conversely, non-hypertensive females had a 31.40% increase in NAFLD risk (OR = 1.314; 95% CI: 1.143–1.511), with both interactions showing statistically significant differences (*p* < 0.01).

Tables 4, 5 provide detailed results of these subgroup analyses.

**Table 4:** Subgroup analysis of serum Mn and NAFLD in male.

Characteristics	OR(95%Cl)	*p* value
Ethnicity		
Mexican American	1.450 (0.950–2.215)	0.085
Other	0.916 (0.730–1.151)	0.453
Non-Hispanic White	1.119 (0.903–1.387)	0.303
Non-Hispanic Black	1.529 (1.164–2.008)	0.002
Education		
Below High School	1.703 (1.233–2.351)	0.001
High School	1.122 (0.876–1.439)	0.362
College or Above	1.052 (0.879–1.261)	0.579
Marital Status		
Married/Living with Partner	1.158 (0.971–1.381)	0.102
Never Married	1.107 (0.858–1.429)	0.435
Widowed/Divorced/Separated	1.295 (0.917–1.828)	0.142
Age		
18–39 Years	1.087 (0.878–1.345)	0.444
40–60 Years	1.193 (0.901–1.579)	0.218
> 60 Years	1.219 (0.977–1.520)	0.079
FMPIR		
< 1.30	1.308 (1.025–1.670)	0.031
1.30 ≤ FMPIR <3.50	1.114 (0.897–1.383)	0.328
≥ 3.50	1.149 (0.909–1.453)	0.245
BMI		
< 25 kg/m^2^	1.146 (0.986–1.332)	0.076
≥ 30 kg/m^2^	1.136 (0.987–1.308)	0.075
25 -< 30 kg/m^2^	1.143 (0.986–1.324)	0.076
Lifetime Smoking Number		
< 100 Cigarettes	1.144 (0.957–1.367)	0.140
≥ 100 Cigarettes	1.173 (0.972–1.416)	0.096
Alcohol Consumption		
Yes	1.181 (1.032–1.351)	0.016
No	0.850 (0.510–1.416)	0.533
Diabetes		
Yes	1.238 (0.810–1.891)	0.323
No	1.151 (1.002–1.322)	0.046
Hypertension		
Yes	1.197 (0.785–1.824)	0.404
No	1.167 (1.015–1.342)	0.030
Hyperlipidemia		
Yes	0.776 (0.498–1.211)	0.264
No	1.211 (1.054–1.391)	0.007

**Table 5:** Subgroup analysis of serum MN and NAFLD in female.

Characteristics	OR (95%Cl)	*p* value
Ethnicity		
Mexican American	1.232 (0.856–1.774)	0.261
Other	1.657 (1.285–2.138)	<0.001
Non-Hispanic White	1.198 (0.989–1.451)	0.065
Non-Hispanic Black	1.239 (0.999–1.537)	0.052
Education		
Below High School	1.565 (1.121–2.184)	0.009
High School	1.330 (1.129–1.566)	0.052
College or Above	1.330 (1.129–1.566)	0.001
Marital Status		
Married/Living with Partner	1.465 (1.225–1.753)	<0.001
Never Married	1.519 (1.140–2.023)	0.004
Widowed/Divorced/Separated	1.056 (0.843–1.323)	0.633
Age		
18–39 Years	1.810 (1.444–2.268)	<0.001
40–60 Years	1.093 (0.874–1.368)	0.436
60+ Years	1.174 (0.950–1.451)	0.137
FMPIR		
< 1.30	1.399 (1.119–1.748)	0.003
1.30 ≤ FMPIR <3.50	1.368 (1.113–1.682)	0.003
≥ 3.50	1.271 (1.000–1.615)	0.050
BMI		
< 25 kg/m^2^	1.306 (0.999–1.539)	0.052
≥ 30 kg/m^2^	1.266 (1.106–1.449)	0.001
25 -< 30 kg/m^2^	1.278 (1.110–1.471)	0.001
Smoking		
Lifetime Smoking ≥ 100 Cigarettes	1.164 (0.94–1.441)	0.163
Lifetime Smoking < 100 Cigarettes	1.438 (1.231–1.68)	<0.001
Alcohol Consumption		
Yes	1.282 (1.120–1.467)	<0.001
No	1.719 (1.207–2.449)	0.003
Diabetes		
Yes	1.016 (0.658–1.567)	0.944
No	1.385 (1.212–1.583)	<0.001
Hypertension		
Yes	1.658 (1.153–2.386)	0.006
No	1.314 (1.143–1.511)	<0.001
Hyperlipidemia		
Yes	1.177 (0.615–2.252)	0.622
No	1.356 (1.191–1.544)	<0.001

## Discussion

Based on the NHANES 2017–2018 survey data, this study found that serum Mn levels in the NAFLD group were significantly higher than those in the control group (*p* = 0.005). After constructing multivariate logistic regression models and adjusting for confounders, serum Mn levels were positively associated with NAFLD in both males and females (male Q3 vs. Q1: OR = 1.575, 95% CI: 1.193–2.087; female Q4 vs. Q1: OR = 1.725, 95% CI: 1.313–2.267).

The restricted cubic spline regression revealed a nonlinear dose–response relationship in males, where Mn acted protectively at lower concentrations (≤8.747 μg/L) but transitioned to a risk factor at intermediate levels (8.747–10.909 μg/L). In contrast, females exhibited a linear relationship, with Mn becoming a risk factor above 9.850 μg/L. These gender-specific patterns may stem from differences in Mn metabolism, hormonal influences, or genetic factors. For instance, estrogen has been shown to modulate Mn homeostasis by affecting transporters like SLC30A10, which regulates Mn excretion (20). Additionally, males may experience higher oxidative stress due to lower baseline antioxidant enzyme activity (e.g., Mn-SOD), amplifying Mn’s toxic effects at intermediate levels (21).

The gender disparity in dose–response relationships could also arise from differences in body composition (e.g., higher lean mass in males altering Mn distribution) or sex-specific expression of Mn-binding proteins (22).

Mechanistically, Mn’s dual role—as a nutrient and toxin—may explain the observed thresholds. At physiological levels, Mn supports mitochondrial function and antioxidant defense via Mn-SOD (23). However, beyond optimal levels, Mn’s toxicity may outweigh its beneficial effects, contributing to the pathogenesis of NAFLD through mechanisms such as increased oxidative stress and inflammation (24, 25).

Notably, conflicting findings from prior studies warrant discussion. While animal models report lower hepatic Mn levels in NAFLD (26), this discrepancy may reflect species-specific Mn metabolism or compensatory mechanisms in chronic disease. Serum Mn levels may not fully correlate with tissue accumulation in advanced NAFLD, as hepatic damage could impair Mn storage or increase systemic release (23). Furthermore, human studies using serum Mn (27) versus tissue-specific measurements (26) may yield divergent results. For example, serum Mn elevation in NAFLD could indicate dysregulated excretion (e.g., via bile) rather than tissue overload (28), a hypothesis requiring validation through paired serum and liver biopsy studies.

Subgroup analyses highlighted interactions between Mn and sociodemographic/clinical factors. In males, ethnicity and education modified Mn-NAFLD associations, possibly due to environmental or occupational Mn exposure disparities (e.g., non-Hispanic Black individuals facing higher industrial pollution). Hypertensive females exhibited stronger Mn-NAFLD links, suggesting shared pathways between Mn toxicity, oxidative stress, and endothelial dysfunction.

Limitations of this cross-sectional study preclude causal inferences. While serum Mn levels are a practical biomarker, they may not fully reflect hepatic Mn accumulation or long-term exposure. Prospective cohort studies with repeated Mn measurements and tissue-level data (e.g., liver biopsies) are needed to clarify causality. Additionally, confounding by unmeasured factors (e.g., dietary Mn intake, genetic polymorphisms in Mn transporters) and recall bias in self-reported covariates (e.g., alcohol use) may influence results.

In conclusion, this study underscores serum Mn as a potential risk factor for NAFLD, with gender-specific thresholds and mechanisms. Future research should prioritize elucidating Mn’s tissue-specific dynamics, longitudinal associations, and molecular pathways in NAFLD pathogenesis. Clinically, monitoring serum Mn in high-risk populations (e.g., industrial workers) and addressing gender-specific risk profiles could enhance NAFLD prevention strategies.

## Data Availability

The original contributions presented in the study are included in the article/supplementary material, further inquiries can be directed to the corresponding author.

## Footnotes

^1^ https://www.cdc.gov/nchs/nhanes/

## References

[1] Byrne CD Targher G. NAFLD: a multisystem disease. J Hepatol. (2015) 62:S47–64. doi: 10.1016/j.jhep.2014.12.012, PMID: 25920090

[2] Younossi ZM Koenig AB Abdelatif D Fazel Y Henry L Wymer M. Global epidemiology of nonalcoholic fatty liver disease-Meta-analytic assessment of prevalence, incidence, and outcomes. Hepatology. (2016) 64:73–84. doi: 10.1002/hep.28431, PMID: 26707365

[3] Della Torre S. Non-alcoholic fatty liver disease as a canonical example of metabolic inflammatory-based liver disease showing a sex-specific prevalence: relevance of estrogen signaling. Front Endocrinol. (2020) 11:572490. doi: 10.3389/fendo.2020.572490, PMID: 33071979 PMC7531579

[4] Le Magueresse-Battistoni B. Endocrine disrupting chemicals and metabolic disorders in the liver: what if we also looked at the female side? Chemosphere. (2021) 268:129212. doi: 10.1016/j.chemosphere.2020.129212, PMID: 33359838

[5] Estes C Razavi H. Modeling the epidemic of nonalcoholic fatty liver disease demonstrates an exponential increase in burden of disease. Hepatology. (2018) 67:123–33. doi: 10.1002/hep.29466, PMID: 28802062 PMC5767767

[6] Argo CK Caldwell SH. Epidemiology and natural history of non-alcoholic steatohepatitis. Clin Liver Dis. (2009) 13:511–31. doi: 10.1016/j.cld.2009.07.005, PMID: 19818302

[7] Li L Yang X. The essential element manganese, oxidative stress, and metabolic diseases: links and interactions. Oxidative Med Cell Longev. (2018) 2018:7580707. doi: 10.1155/2018/7580707, PMID: 29849912 PMC5907490

[8] Al-Serri A Anstee QM Valenti L Nobili V Leathart JB Dongiovanni P. The SOD2 C47T polymorphism influences NAFLD fibrosis severity: evidence from case-control and intra-familial allele association studies. J Hepatol. (2012) 56:448–54. doi: 10.1016/j.jhep.2011.05.029, PMID: 21756849

[9] Mota M Banini BA Cazanave SC Sanyal AJ. Molecular mechanisms of lipotoxicity and glucotoxicity in nonalcoholic fatty liver disease. Metab Clin Exp. (2016) 65:1049–61. doi: 10.1016/j.metabol.2016.02.014, PMID: 26997538 PMC4931958

[10] Rezazadeh A Yazdanparast R. Prevention of nonalcoholic steatohepatitis in rats by two manganese-salen complexes. Iran Biomed J. (2014) 18:41–8. doi: 10.6091/ibj.1201.2013, PMID: 24375162 PMC3892139

[11] Hyder O Chung M Cosgrove D Herman JM Li Z Firoozmand A. Cadmium exposure and liver disease among US adults. J Gastrointest Surg. (2013) 17:1265–73. doi: 10.1007/s11605-013-2210-9, PMID: 23636881 PMC3974907

[12] Tan M Schmidt RH Beier JI Watson WH Zhong H States JC. Chronic subhepatotoxic exposure to arsenic enhances hepatic injury caused by high fat diet in mice. Toxicol Appl Pharmacol. (2011) 257:356–64. doi: 10.1016/j.taap.2011.09.019, PMID: 21983427 PMC3232462

[13] Cave M Appana S Patel M Falkner KC McClain CJ Brock G. Polychlorinated biphenyls, lead, and mercury are associated with liver disease in American adults: NHANES 2003-2004. Environ Health Perspect. (2010) 118:1735–42. doi: 10.1289/ehp.1002720, PMID: 21126940 PMC3002193

[14] O'Neal SL Zheng W. Manganese toxicity upon overexposure: a decade in review. Curr Environ Health Rep. (2015) 2:315–28. doi: 10.1007/s40572-015-0056-x, PMID: 26231508 PMC4545267

[15] Johnson CL Paulose-Ram R Ogden CL Carroll MD Kruszon-Moran D Dohrmann SM. National health and nutrition examination survey: analytic guidelines, 1999-2010 In: Vital and health statistics Series 2, Data evaluation and methods research, 161 (2013). 1–24. 25090154

[16] Zhang K Nulali J Zhang C Chen Y Cheng J Shi X. The association between serum vitamin a and NAFLD among US adults varied in different BMI groups: a cross-sectional study. Food Funct. (2023) 14:836–44. doi: 10.1039/D2FO02204D, PMID: 36321945

[17] Peng H Pan L Ran S Wang M Huang S Zhao M. Prediction of MAFLD and NAFLD using different screening indexes: a cross-sectional study in U.S. adults. Front Endocrinol. (2023) 14:1083032. doi: 10.3389/fendo.2023.1083032, PMID: 36742412 PMC9892768

[18] Wang X Seo YA Park SK. Serum selenium and non-alcoholic fatty liver disease (NAFLD) in U.S. adults: National Health and nutrition examination survey (NHANES) 2011-2016. Environ Res. (2021) 197:111190. doi: 10.1016/j.envres.2021.111190, PMID: 33872646 PMC8187321

[19] Williams M Todd GD Roney N Crawford J Coles C McClure PR. Agency for Toxic Substances and Disease Registry (ATSDR) toxicological profiles In: Toxicological profile for manganese. Atlanta (GA): Agency for Toxic Substances and Disease Registry (US) (2012) 24049862

[20] Kochhar S Jacobs DM Ramadan Z Berruex F Fuerholz A Fay LB. Probing gender-specific metabolism differences in humans by nuclear magnetic resonance-based metabonomics. Anal Biochem. (2006) 352:274–81. doi: 10.1016/j.ab.2006.02.033, PMID: 16600169

[21] Sabato V Ebo DG Koppelman SJ Jayasena S Luykx D Schepens E. Allergenicity attributes of different peanut market types. Food and chemical toxicology: an international journal published for the British industrial biological research association 2016. Food Chem Toxicol. (2016) 92:256. doi: 10.1016/j.fct.2016.03.019, PMID: 26921497

[22] Marchetti A Orlando M. A cold-active esterase enhances mesophilic properties through Mn (2+) binding. FEBS J. (2023) 290:2394–411. doi: 10.1111/febs.16661, PMID: 36266734

[23] Tang S Luo S Wu Z Su J. Association between blood heavy metal exposure levels and risk of metabolic dysfunction associated fatty liver disease in adults: 2015-2020 NHANES large cross-sectional study. Front Public Health. (2024) 12:1280163. doi: 10.3389/fpubh.2024.1280163, PMID: 38435294 PMC10904630

[24] Santamaria AB. Manganese exposure, essentiality & toxicity. Indian J Med Res. (2008) 128:484–500. PMID: 19106442

[25] Horning KJ Caito SW Tipps KG Bowman AB Aschner M. Manganese is essential for neuronal health. Annu Rev Nutr. (2015) 35:71–108. doi: 10.1146/annurev-nutr-071714-034419, PMID: 25974698 PMC6525788

[26] Gatiatulina ER Popova EV Polyakova VS Skalnaya AA Agletdinov EF Nikonorov AA. Evaluation of tissue metal and trace element content in a rat model of non-alcoholic fatty liver disease using ICP-DRC-MS. J Trace Elem Med Biol. (2017) 39:91–9. doi: 10.1016/j.jtemb.2016.08.007, PMID: 27908430

[27] Wan H Jiang Y Yang J Ma Q Liu L Peng L. Sex-specific associations of the urinary fourteen-metal mixture with NAFLD and liver fibrosis among US adults: a nationally representative study. Ecotoxicol Environ Saf. (2022) 248:114306. doi: 10.1016/j.ecoenv.2022.114306, PMID: 36402077

[28] Wu T Yang M Xu H Wang L Wei H Ji G. Serum bile acid profiles improve clinical prediction of nonalcoholic fatty liver in T2DM patients. J Proteome Res. (2021) 20:3814–25. doi: 10.1021/acs.jproteome.1c00104, PMID: 34043368

